# Mesoporous Bioactive Glasses Cytocompatibility Assessment: A Review of In Vitro Studies

**DOI:** 10.3390/biomimetics6010009

**Published:** 2021-01-23

**Authors:** Margaux Salètes, Marta Vartin, Caroline Mocquot, Charlène Chevalier, Brigitte Grosgogeat, Pierre Colon, Nina Attik

**Affiliations:** 1 CPE Lyon, Université Claude Bernard Lyon 1, CEDEX 08, 69372 Lyon, France; margaux.saletes@cpe.fr (M.S.); marta.vartin@cpe.fr (M.V.); 2 Laboratoire des Multimatériaux et Interfaces, UMR CNRS 5615, Université de Lyon—Université Claude Bernard Lyon 1, CEDEX 08, 69372 Lyon, France; caroline.mocquot@etu.univ-lyon1.fr (C.M.); charlene.chevalier@univ-lyon1.fr (C.C.); brigitte.grosgogeat@univ-lyon1.fr (B.G.); pierre.colon@univ-paris-diderot.fr (P.C.); 3 Assistance Publique-Hôpitaux de Paris, Hôpital Rothschild, Service D’odontologie, Faculté Dentaire, Université de Paris, 75012 Paris, France; 4 Faculté d’Odontologie, Université de Lyon, Université Claude Bernard Lyon 1, 69008 Lyon, France; 5 Hospices Civils de Lyon, Service D’odontologie, 69007 Lyon, France

**Keywords:** mesoporous bioactive glasses, in vitro, cytocompatibility, bioactivity, medical applications

## Abstract

Thanks to their high porosity and surface area, mesoporous bioactive glasses (MBGs) have gained significant interest in the field of medical applications, in particular, with regards to enhanced bioactive properties which facilitate bone regeneration. The aim of this article is to review the state of the art regarding the biocompatibility evaluation of MBGs and provide a discussion of the various approaches taken. The research was performed using PubMed database and covered articles published in the last five years. From a total of 91 articles, 63 were selected after analyzing them according to our inclusion and exclusion criteria. In vitro methodologies and techniques used for biocompatibility assessment were investigated. Among the biocompatibility assessment techniques, scanning electron microscopy (SEM) has been widely used to study cell morphology and adhesion. Viability and proliferation were assessed using different assays including cell counting and/or cell metabolic activity measurement. Finally, cell differentiation tests relied on the alkaline phosphatase assay; however, these were often complemented by specific bimolecular tests according to the exact application of the mesoporous bioactive glass. The standardization and validation of all tests performed for MBG cytocompatibility is a key aspect and crucial point and should be considered in order to avoid inconsistencies, bias between studies, and unnecessary consumption of time. Therefore, introducing standard tests would serve an important role in the future assessment and development of MBG materials.

## 1. Introduction

Bioactive glasses (BGs) represent a major discovery in tissue repair. The first BG ever synthesized was the 45S5 Bioglass^®^ in 1969 by Professor Larry Hench and was a clinical success for cochlear bone repair [1]. It marked the beginning of the development from bio-inert materials towards bioactive materials that have the property of interacting closely with tissues to drive regeneration [2].

BG’s bioactivity is attributed to the two following properties [3]:

- The formation of a biomimetic hydroxyapatite layer after glass immersion after interaction with biological fluids;
- The osteogenic ability of some dissolution products and leachable compounds and ions.

From then, the BG technology was well developed, allowing researchers to elaborate new materials, such as BG composites, nanobioactive glasses, doped-BGs, mesoporous bioactive glasses (MBGs) [3].

Nowadays, due to their ease of synthesis and remarkable properties, BGs have found uses in a wide range of medical applications, for example, bone regeneration [4], prosthesis and implant coating [5,6], and hemostasis [7]. In dentistry, BG-based products are also valuable for many clinical indications, such as dental hypersensitivity treatment [8]. A classification of methodologies for BG bioactivity quantification has been recently established, aimed to a better understanding of the bioactive properties of BG regarding dental hard tissues [9].

**Figure 1:**
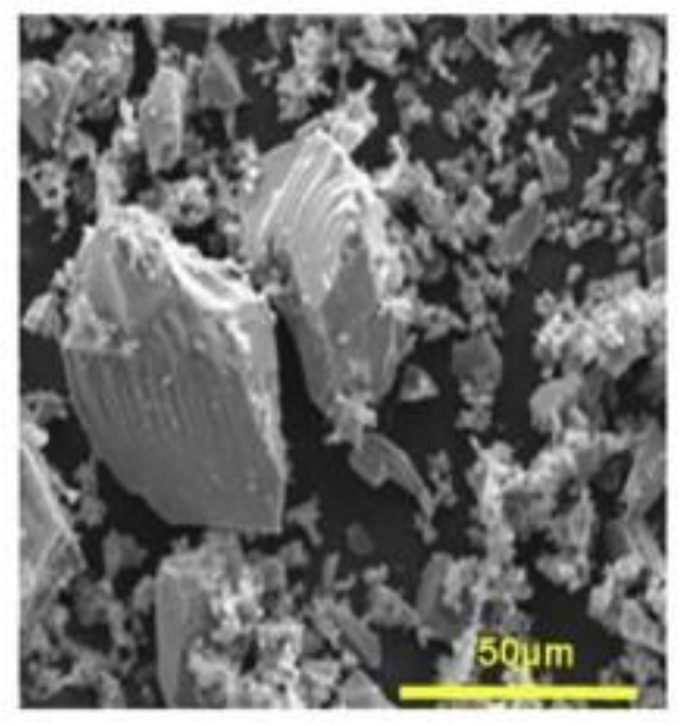
Surface of a mesoporous bioactive glass [11]. Scale bar: 50 μm.

The current review focuses on MBG-based materials which were first introduced in 2004 [10]. After the melt quench technique, the sol-gel method is the most opted way to prepare such glasses. Use of a bottom-up, low temperature synthesis route with versatile process parameters offers the control needed to achieve particles with enhanced features. Briefly, this process consists of creating a gel by polycondensation of silicon alkoxides Si(OR)_4_ in an aqueous phase. When a surfactant is introduced to the process, it tends to form three dimensional micellular structures within the gel. The gel is then calcined up to 700 °C to eliminate the organic components, leaving only the glass and its “cavities” (Figure 1) [11].

This specific method gives the mesoporous glass an intrinsic porosity and a very high specific surface area of hundreds of m^2^/g, unlike the conventional fusion method that leads to surface areas of few m^2^/g. High porosity and specific surface area positively influence the bioactivity through accelerated dissolution of ions from the glass [12,13,14]. The mesoporous aspect of these glasses was first used for catalytic applications [15]. However, it soon became an essential engineering technique for materials as it was possible to control the porosity by modifying the process parameters. In fact, the size of the pores can even reach the micrometer scale using opal prints instead of surfactants [16] which offers new perspectives for the delivery of high molecular weight molecules [17]. The porosity of MBG has been demonstrated as leading to particular efficiency in promoting remineralization for bone regeneration [18,19]. Furthermore, MBG can be doped with different elements to attain new functionalities, such as, Cu-doped MBG which has been reported to have angiogenic and antibiotic effects [20].

MBG-based materials seem to be very promising for medical applications, including in dentistry, and, thus, a significant amount of research has recently been conducted with regards to their effect on the remineralization of calcified dental tissues and the dentin-pulp complex tissues. More recently, Mocquot et al. revealed an enhancement of the metabolic activity and the mineralization ability of primary dental pulp cells when they were subjected to MBG-based particles [21].

As MBGs represent a new generation of conventional bioactive glasses with unique properties (high surface area and high porosity) and have the potential for use across a large spectrum of medical applications, important questions should be addressed concerning the evaluation of their biocompatibility. Since the prime focus of researchers is the development of improved bioactive glasses that are mainly safe and suitable for industrial and clinical use, the aim of this review was therefore to identify and rank the different methods used for the MBG biocompatibility assessment in vitro.

## 2. Methods

### 2.1. Research Question

Before any literature investigation, the following research question had been formulated: “How to assess the in vitro biocompatibility of MBG?”

### 2.2. Search Strategy

PubMed was the only electronic database used for this review. The scientific articles published from July 2015 to November 2020 were automatically investigated.

### 2.3. Keywords Selection

Two different keyword combinations were tested and the one resulting with the maximum number of articles was selected.

### 2.4. Inclusion and Exclusion Criteria

To determine the relevant studies needed to conduct the current research review, inclusion and exclusion criteria were established.

The articles were included according to the following rules:

(1) Research article;
(2) Use of mesoporous bioactive glasses with and without scaffolds or composite;
(3) Detailed investigation of biocompatibility in vitro.

On the other hand, the following were systematically removed from our study:

(1) Review articles;
(2) Articles that used non-mesoporous bioactive glasses;
(3) Articles that did not explain their biocompatibility methods;
(4) Articles that did not carry out biocompatibility tests in vitro.

### 2.5. Paper Selection and Data Extraction

Each article abstract was carefully read by the two first authors MS and MV to determine whether or not the article should be included in the study. Once the articles to be included were shortlisted, their full text was then read and investigated to explore different parameters:

- Medical application of the MBG;
- MBG composition and synthesis pathway;
- Cells used for biocompatibility tests;
- Biocompatibility tests performed and their results.

## 3. Results

In view of the small number of articles referring to the dental field, it was decided that the scope should be extended to also assess the biocompatibility of MBGs used for different medical applications. The search equation retained was as follows: “((mesoporous) AND (bioactive) AND (glass)) OR (MBG) AND (biocompatibility)” as it returned the maximum number of articles (Table 1).

**Figure 2:**
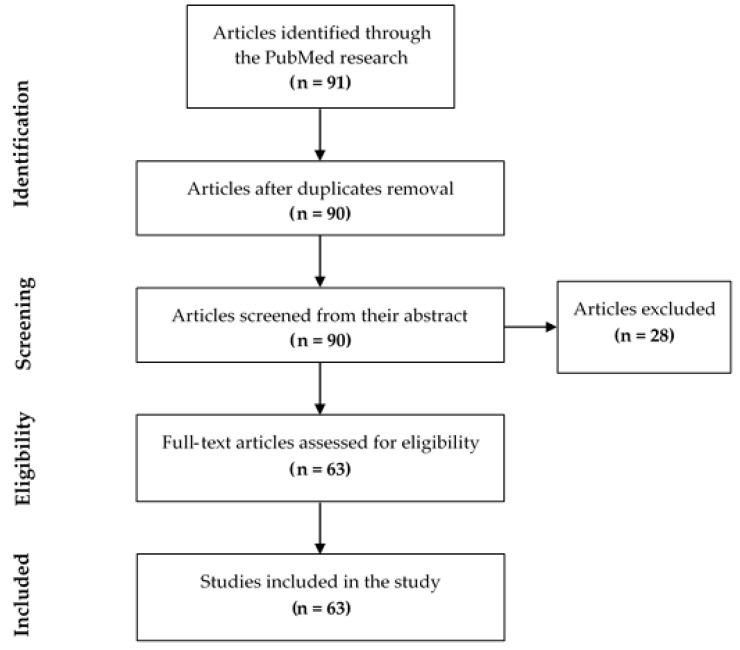
Flow diagram of study identification.

Duplicate articles were excluded (one article). Then, the title and abstract of each of the remaining 90 articles were examined allowing the exclusion of 28 articles that did not meet the inclusion criteria (Figure 2). The exclusion reason for each article is described in Table 2. Finally, 63 relevant articles were investigated by reading the full text (Table 3).

### 3.1. Control Group Used to Compare the Biocompatibility of Mesoporous Bioactive Glasses (MBGs)

A choice of suitable control groups provides the basis on which the relative performance of the materials can be compared. This choice is therefore fundamental to each study to affirm or refute differences in the effects of the MBG studied. The vast majority of articles describe the use of an equivalent MBG (same composition) as the control group but without doping and/or adjuvant and/or functionalization.

About 6% (four articles [71,77,85,102]) of MBGs used a conventional BG as the control group. Mainly, MBGs show a significantly better biocompatibility than conventional BGs. MBG scaffolds demonstrate notably better cell viability and differentiation [77]. The use of negative controls could also be of interest in order to establish the normal behavior of cells under the experimental conditions of each protocol.

### 3.2. Study Characteristics

#### 3.2.1. MBG Synthesis, Characteristics, and Application Areas

The sol-gel process is a wet-chemistry technique widely used in the production of vitreous and ceramic materials. This synthesis technique allows control over the morphology (nano-, micro-, and macro-size) and composition of the resulting mesoporous materials by varying the process parameters or by carrying out additional treatment at the gel stage of the materials [109]. One of the advantages of the sol-gel method is the simplicity of the equipment necessary for synthesis.

Nevertheless, and in view of green chemistry, the sol-gel process for bioactive glasses synthesis requires inorganic acids as catalysts, which have adverse effects on health and the environment [110]. Recently, Dang et al. used a modified sol-gel method to successfully elaborate bioactive glasses with the composition of 70SiO_2_-30CaO (mol.%) and with no acid catalysts [111]. With regard to environmental protection and human health, environmentally friendly methods for mesoporous bioactive glasses elaboration are required following the trend of green chemistry.

**Figure 3:**
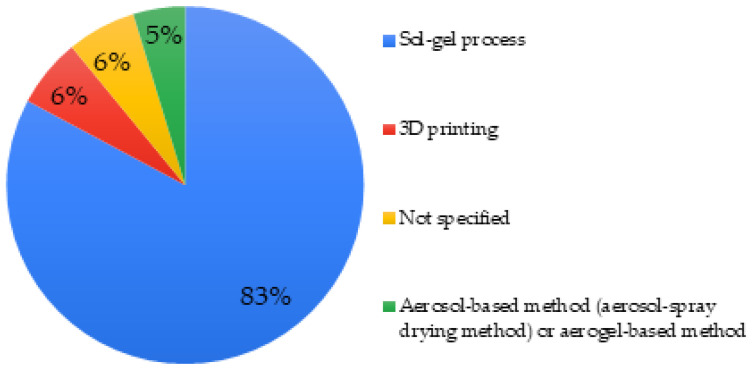
Mesoporous bioactive glass (MBG) synthesis methods used in studied articles.

Eighty-three percent of the MBGs studied were synthesized by the sol-gel process. More rarely, a synthesis assisted by an aerosol or aerogel method was reported (5%), making it possible to produce ordered mesoporous microspheres with a high degree of sphericity and an ordered mesostructure [112]. These different techniques were found in articles describing micro- or nano-MBGs [66,69]. In general, the sol-gel process was found to be employed for all sizes and morphologies of MBG. Finally, in 6% of cases, MBGs were synthesized by 3D printing via so-called additive manufacturing (Figure 3).

Regarding the basic composition of MBG used, the great part of MBG used have a basic composition consisting of SiO_2_-CaO-P_2_O_5_ with variable proportions. These relative proportions regulate parameters such as surface area, pore volume, and pore size, and thus consequently influence the mesoporous properties of MBG [113].

**Figure 4:**
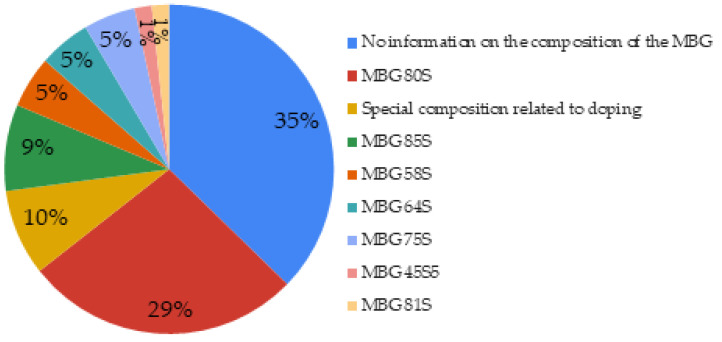
Basic compositions of MBGs used in the studied articles.

The composition of the MBG was not specified in 35% of cases, even in the associated references; in such cases, the assessment or the comparison of any effect of composition cannot be discussed. The compositions which returned most often (29%) were the MBG 80S15C (80SiO_2_: 15CaO: 5P_2_O_5_) and 80S16C (80SiO_2_: 16CaO: 4P_2_O_5_). More rarely, specific compositions related to doping (10%) or MBG compositions 85S15C (85SiO_2_: 15CaO) were found (Figure 4).

MBGs have several established medical applications such as bone regeneration [18], dentin remineralization [114], and much more. Studies are also underway to use mesoporous bioactive glasses for dental applications such as the prevention of prosthetic joint infections [115], as fillers in restorative materials, in direct contact with dentin and/or pulp tissues [21], dentinal sealing for the treatment of dentin hypersensitivity [116], coating for dental implants [115], and the treatment of periodontal diseases [117]. From the studies included, 74% of articles used MBG for bone regeneration. More rarely, the applications were drug delivery, osteoporosis, or hemostasis. Gene delivery is also observed as one of the application areas of MBG providing a mechanism for the introduction of foreign genetic material (DNA, RNA, etc.) into host cells. This material will then migrate into the nucleus of the host cells and could induce modification on the gene expression profile. From another perspective, Zhou et al. assessed the inflammatory profile of some MBG nanoparticles incorporated in hybrid scaffolds using immunity cells [108].

#### 3.2.2. Doping and Adjuvants of Studied MBGs

Due to high porosity, MBGs can be doped or they can be mixed with adjuvants like polymers to gain new properties [20]. It is also possible to ensure drug delivery [17].

**Figure 5:**
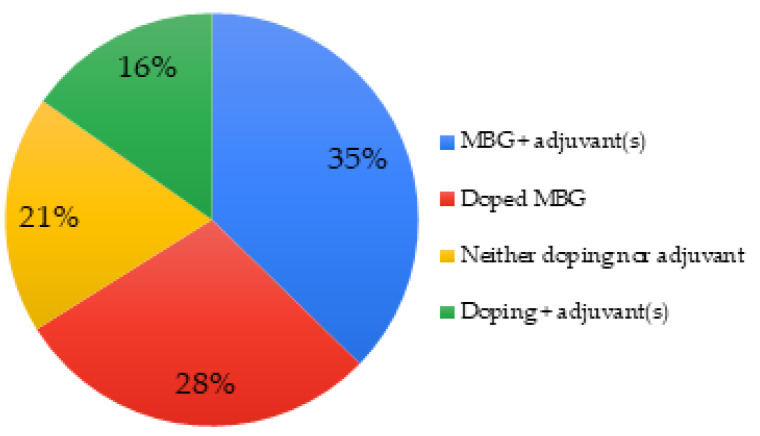
Doping and adjuvants of MBGs used in studied articles.

The porosity of mesoporous bioactive glasses was exploited in 51% of the articles by incorporating adjuvants inside the pores, such as growth factors [62]. It is also common to add them to polymers to create composites, for example, polycaprolactone [90,95]. In 28% of the cases, the MBGs were doped, either with a metal-like element or with amino groups. Almost systematically, adding a dopant decreases the level (mol.%) of silicon in the MBG [55], see Figure 5.

In general terms, MBGs offer suitable platforms for drug/ion delivery which increases the range of possible uses in the biomedical field. For example, incorporation of strontium for the treatment of osteoporosis, cobalt to enhance the pro-angiogenic effects, and copper to improve angiogenesis and immune responses [118,119].

### 3.3. Cells Characteristics

**Figure 6:**
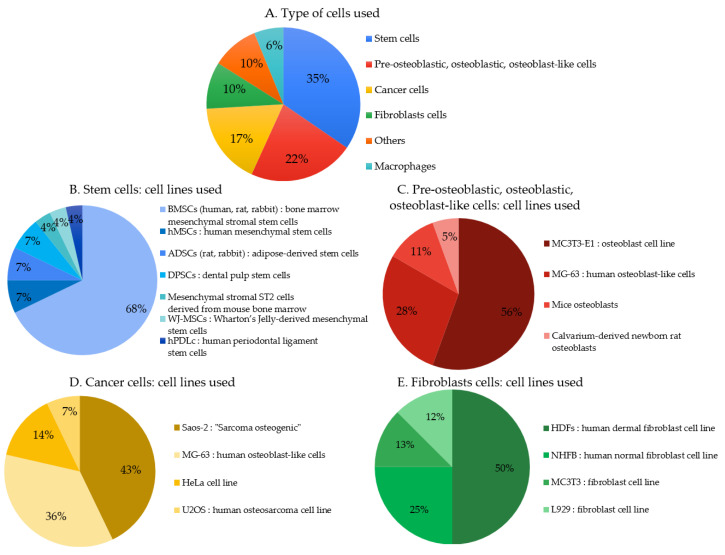
(**A**–**E**). Primary cells and cell lines used to assess cytocompatibility in the studied articles.

Twenty-eight articles indicate the use of stem cells (Figure 6A) to perform biocompatibility tests in which about 3/4 were BMSCs (bone marrow mesenchymal stem cells) derived from different mammals (human, rat, rabbit) (Figure 6B). Eighteen articles indicate the use of pre-osteoblastic, osteoblastic, or osteoblast-like cells (mostly from animal origin). Fifty-six percent used MC3T3-E1 type cells and 28% used cells from MG-63 lines (Figure 6C). Fourteen articles indicate the use of cancer cells (Figure 6A); among them 43% were Saos-2 (“Sarcoma osteogenic”) (Figure 6D). Eight articles indicate the use of fibroblasts cells, half were HDFs (human dermal fibroblast) cells (Figure 6E). From a clinical perspective, the cell type should be carefully chosen according to the targeted medical application.

### 3.4. Techniques Used to Assess the MBG In Vitro Biocompatibility

#### 3.4.1. Cell Morphology

Morphological changes provide a first indication of the behavior of cells in contact with MBGs and can be studied using a variety of different techniques.

**Figure 7:**
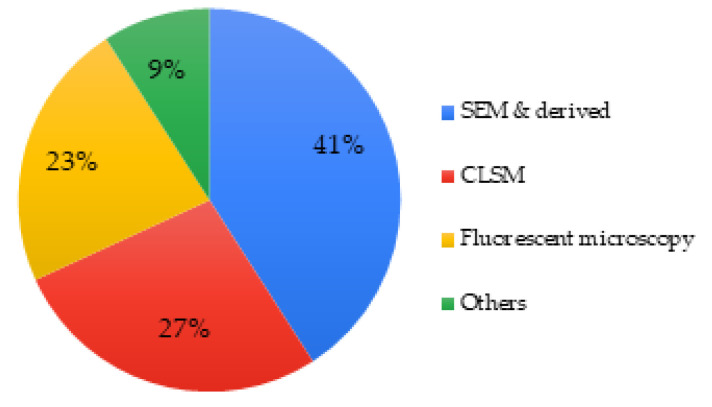
Techniques used to assess cell morphology of MBGs.

About 27% of articles investigated the modifications of cell morphology. Microscopy was the technique used by all, for example, scanning electron microscopy (SEM) was the primary choice, followed by confocal laser scanning microscopy (CLSM) and fluorescence microscopy, which was used in a lesser proportion of studies. Transmission electron microscopy (TEM) and inverted optical microscopy were also found to a lesser extent (Figure 7).

#### 3.4.2. Cell Adhesion/Attachment

Cell adhesion/attachment is a dynamic process resulting from specific interactions between cell surface molecules and appropriate ligands [120] which is studied by imaging techniques. It is an essential parameter to validate the biocompatibility of a biomaterial.

**Figure 8:**
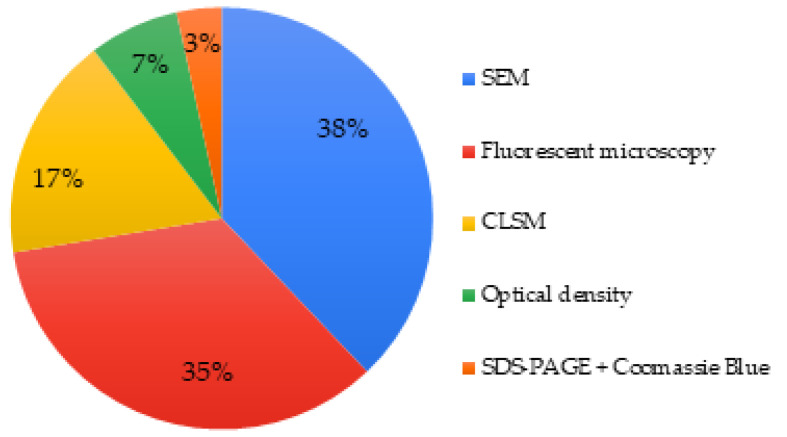
Techniques used to assess cell adhesion/attachment of MBGs.

About 38% articles investigated the cell adhesion/attachment on the MBG surface. Once again, microscopy was a technique commonly used representing 90% of the studies. SEM and fluorescence microscopy were the most common. Optical density and SDS-PAGE combined with Coomassie Blue visualization were explored as alternative options in two separate articles (Figure 8) [49,105].

#### 3.4.3. Cell Viability and Proliferation

First of all, a cell viability assay is performed to assess the proportion of healthy and viable cells within a population. In a cell proliferation assay, the result is a precise measurement of the number of cells dividing [121]. A significant number of articles have confused the notions of cell viability, cell proliferation, and general cytotoxicity. The data have been reclassified clearly for this review.

**Figure 9:**
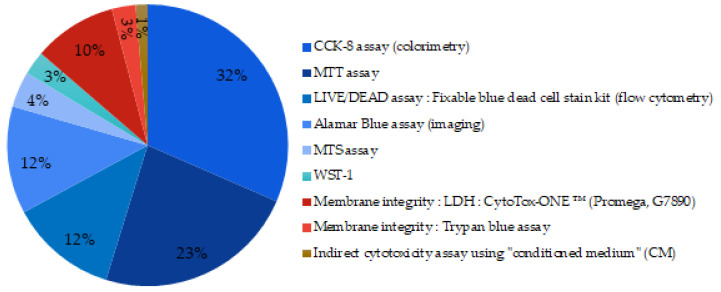
Techniques used to assess cell viability of MBGs. (blue part: metabolic assays; red part: membrane integrity measurement).

About 94% of articles investigated the cell viability and/or cell proliferation. A large panel of techniques were found for evaluating these parameters (Figure 9).

Eighty-six percent of the tests used were metabolic based assays. They are illustrated by the shaded blue portions in the graph (Figure 9). CCK-8 (Cell Counting Kit-8) assay uses a salt further reduced by active metabolic cells via dehydrogenase enzymes. The reduction product obtained is colored and its concentration is determined by colorimetric measurements. It is by far the technique most widely reported in the studies. The MTT (3-(4,5-dimethylthiazol-2-yl)-2,5-diphenyltetrazolium bromide) assay and the Alamar Blue assay that require different enzymes and reagents but follow the same oxide-reduction reaction were also reported. The LIVE/DEAD staining was used in 12% of the studies, it is a membrane integrity-based assay, and results are revealed by fluorescence measurement. Pintor et al. compared MTT assay to other cell viability assays and found that MTT and XTT (2,3-bis-(2-methoxy-4-nitro-5-sulfophenyl)-2H-tetrazolium-5-carboxanilide) assays do not induce over- or underestimation of the cell viability and were in at least moderate agreement with other cell viability assays when the root canal filling materials were screened [122].

#### 3.4.4. Apoptosis Quantification, Cell-Cycle Analysis

Only three articles [79,90,104] investigated apoptosis quantification by flow cytometry, and four articles [71,79,90,91] investigated cell-cycle analysis using the same flow cytometry technique.

#### 3.4.5. Cell Differentiation

Biomaterials and scaffolds play an essential role in guiding the target tissue growth, healing, and regeneration. That is why cell differentiation tests are essential for neo-tissue formation and could provide an indication of biomaterial bioactivity.

**Figure 10:**
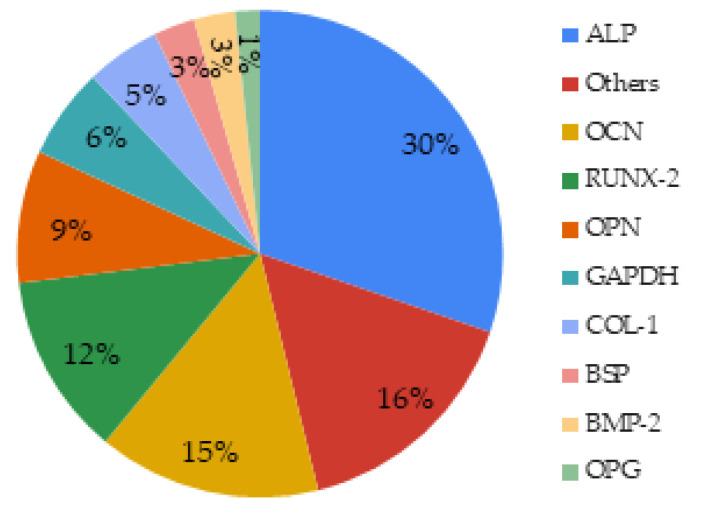
Markers studied to assess the effect of MBGs on cell differentiation.

Forty-four articles (about 70%) investigated cell differentiation. In order to do so, different cell markers had been used. A common method quantifies the alkaline phosphatase (ALP), an enzyme present in all differentiated tissues by colorimetric techniques in most of the investigated studies or by immunological techniques such as ELISA and western blot techniques in few studies [62,99]. Fluorescent or normal staining were also used to visualize ALP production and provided qualitative data [73,97]. qRT-PCR (quantitative reverse transcription PCR) targeting RNA coding for ALP is also a method to study cell proliferation. Other differentiation markers were found, like COL-1 (type I collagen), BMP-2 (bone morphogenetic protein-2), and GAPDH (glyceraldehyde phosphate dehydrogenase) used commonly as a reference gene (Figure 10).

In general terms, cell differentiation could be assessed more clearly in primary and undifferentiated cells due to their sensitivity and their potential for expression of key differentiation markers.

Table 4 lists the main specific tissue markers associated with bone regeneration and angiogenesis; two of the common most themes amongst the articles reviewed.

**Figure 11:**
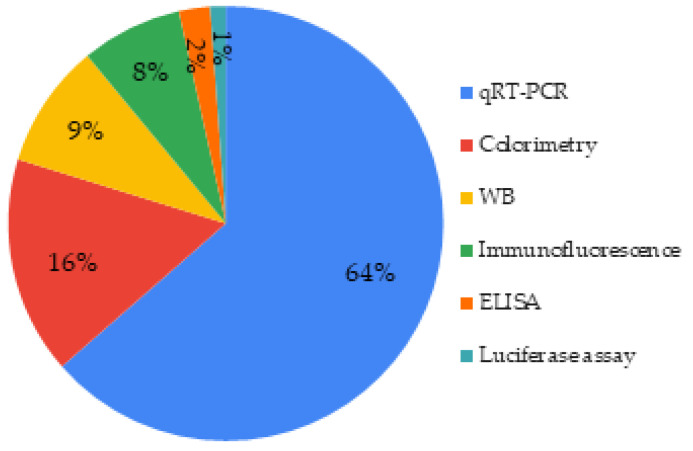
Techniques used to assess cell differentiation.

There are different ways to evaluate and quantify those markers, qRT-PCR being the predominant one (64%). Among immunological techniques in the studies selected, Western Blot (WB) was the most common (Figure 11).

### 3.5. Cell cultivation Setting (Direct and Indirect Contact)

In certain clinical situations, MBGs or MBG-based materials do not make direct contact with cells, however, their dissolution products or leachates could reach targeted cells; this could explain the significant use of extraction techniques (indirect cultivation setting) when assessing cytocompatibility of materials [21,55,59,81,91,95,100,101]. In a test based on direct contact, the material sample is in physical contact with the cells; this system also has some clinical relevance. In this direct cultivation setting, test specimens are placed on top of an established cell monolayer as reported by [82] or on the top of the material surface [80]. According to the intended clinical application, the cultivation setting (direct or indirect) is selected. When direct contact is used, precautions should be taken to maintain the physiological balance of cells, on the other hand, the extraction method used to obtain the tested eluates should be suitable and allow an optimum of leachable materials in the indirect cultivation setting [123].

## 4. Discussion

The vast majority of MBGs studied were synthesized by the sol-gel process (83% of the studies, Figure 3). It is a process also widely used in the synthesis of conventional BGs because it not only allows fabrication of glasses according to different morphologies (fibers, powders, coatings, 3D porous scaffolds) but the synthesis is carried-out at a relatively low temperature with relatively simple equipment (in comparison with other synthetic methods such the melt quench technique). This process also makes it possible to offer a large surface area of active Si-OH sites which can potentially be functionalized for more specific applications [124]. Indeed, the high mesoporosity and surface area of bioglasses prepared by sol-gel techniques enhance the kinetics of apatite formation and expand the compositional range [125]. The choice of synthesis of MBGs by sol-gel method seems to be the most relevant in medical applications because of its simplicity and the ability to tailor properties. Other synthesis methods exist such as the melt quench method or EISA (evaporation induced self-assembly). The first consists of melting silicon oxides at 1300 °C and then cooling the molten glass quickly in a mold. However, high temperature results in the loss of residual surface silanols Si-OH in favor of siloxane bonds Si-O-Si and the loss of particle porosity. Chitra et al. investigated the structural properties of a sol-gel-derived bioactive glass and found that the use of a probe sonication-assisted sol-gel method resulted in enhanced glass porosity and control over particle size [102]. Obtained nanoparticles were mesoporous in nature and exhibited a higher rate of biocompatibility and hydroxylapatite layer precipitation on the surface compared to conventional glass particles and micro glass particles [102]. EISA combines the sol-gel technique and supramolecular chemistry to obtain MBG particles with high surface area [32].

The introduction of 3D printing in the field of BGs, and especially, gel-derived BGs is showing great promise for expanding the applications of these materials. Indeed, fabrication of hierarchical MBG-based scaffolds is a challenge. In fact, mesopore size is almost three orders of magnitude lower than that of osteoblasts and, therefore, macroporosity must be somehow introduced in the final product in order to allow cell colonization and tissue in-growth [126].

Glass bioactivity is also affected by the glass composition. Bioactivity is the property of interacting with a tissue to drive its repair [2]. It is commonly evaluated by an index, I*_B_*, linked to the parameter, t_1/2_, which is half the time necessary for the glass surface to be covered with cells.

$$I_{B}=\;\frac{100}{t_{1/2}}$$

In 38% of MBG whose composition was indicated in the articles, the proportion of SiO_2_ was greater than or equal to 80% mol. This is explained by the increase in the surface area, pore volume, and pore size when the SiO_2_ content increases [113]. The high concentration of SiO_2_ plays a key role in bone metabolism and collagen synthesis. The most frequent composition for MBG comprised SiO_2_:CaO:P_2_O_5_. The porosity increased when calcium oxide increased, compared with sodium oxide [32]. The compositions with high sodium interfered with the textural features by reducing the porosity because of the fusion of pores [10]. Porosity has a significant role at the interface with cellular membranes and could enhance bioactivity as a result of enhanced surface area [38]. Moreover, glasses with CaO, P_2_O_5_, and SiO_2_ in their composition exhibit preferential layer formation on their surface involving the Si-OH group formation including a heterogeneous nucleation of apatite [125].

Thanks to their porosity and their structure, MBGs offer a panel of possibilities in doping and addition of adjuvants according to the target application. These particular MBGs represented about 79% of the articles studied.

Most common dopants in MBGs were gallium, strontium, or copper [20,67,93]. Strontium is known to be a bone-seeking agent, improving stimulation of osteoblasts and having anabolic and anti-catabolic properties. It also has the ability to increase resistance to dissolution [127,128,129] with some antibacterial activity. Copper and gallium, on the other hand, improve biomineralization and amplify antimicrobial properties [67,128,130]. Wu et al. suggested the doping of MBG foams with europium (1–5 mol.%) to fabricate luminescent scaffolds for biolabeling and clinical imaging applications without altering the bioactivity [131]. Europium-doped MBG scaffolds contributed to accelerated bone regeneration via the enhanced stimulation of new bone formation.

MBGs have also been investigated as controlled drug release systems. The combination of excellent surface properties and porosity, as well as the ability to be functionalized, allows them to act as release systems for antibiotics and/or osteogenic agents [132].

The review of Baino et al. focused on bioactive glass-based hierarchical materials and raises the issue of the potential health hazard related to the particle size of mesoporous nano-beads in implantable systems. In fact, silica nanoparticles of different sizes (250 and 500 nm) penetrated into the cells (A549 and RAW264.7 types), being compartmentalized within endocytic vacuoles, and induced genotoxicity [38].

Regarding adjuvants and polymer-based materials, sodium alginate, chitosan, and polycaprolactone were the frequent polymers found in order to synthesize the scaffolds.

We noticed that, in most of these cases, cytotoxicity was directly associated with doping and/or adjuvants in a dose-dependent and time-dependent way. However, the literature reports that the introduction of dopants decreases the surface area and the pore volume of MBG, and consequently significantly decreases its mesoporous properties [113]. Thus, this property of mesoporosity of MBGs represents not only their strength but also their weakness by affecting some of their mechanical properties and the release of mesoporous components of nanometric size after their introduction in the human body [133].

The choices of the control groups are relevant for all of the articles studied. Some articles have gone further by studying the differences between MBGs and conventional BGs. The literature references significantly better biocompatibility results for MBGs compared to conventional BGs [113]. The results of biocompatibility assays emanating from several articles studied in this review draw the same conclusion, indicating that scaffold MBGs can promote osteogenic differentiation [72,79].

For the MBG cytocompatibility assessment, the majority of the investigated studies used different immortalized cell lines (about 75% of the studies) such as MG-63 osteoblasts like cells [80,98]. The osteoblastic phenotype of MG-63 cells is particularly interesting for the study of bone regeneration [92]. Some studies have revealed that there are differences between established immortalized cell lines and primary cells in cellular response to the biomaterials in vitro [134,135]. Although established cell lines have the advantage of being immortal, available worldwide, and easy to grow, they remain distant from target cells and therefore do not allow the study of necessary markers (such as markers of bone remineralization). Primary cell cultures, on the other hand, are cells with a limited lifespan (because they differentiate after a few multiplications), but they are closest to the target tissues. This considerable advantage from primary cell lines makes it possible to study markers which cannot be studied with established cell lines, and to study bone remineralization in a more in-depth and relevant manner.

Regarding the cells, researchers used a large panel of different cell types. However, according to Johnson et al., there is a real difference in sensitivity of established cell lines that can differ even in controlled conditions [136]. The use of primary human cells or established lines of mammals are acceptable only if these cells are of sufficiently high quality to allow cell reproduction throughout the duration of the experiment, while maintaining their phenotypic characteristics. An official guideline could harmonize their use in the biomedical research. Wilkesmann et al. compared the effect of the crystallized 45S5 bioactive glasses on the viability and the osteogenic differentiation of different human osteogenic cells. Their data demonstrated that hOBs (human osteoblasts), BMSCs, and MG-63 cells were resistant to 45S5-BG induced cytotoxicity, while the viability of Saos-2, HOS (*Homo sapiens* bone osteosarcoma cells) and U2OS cells was significantly decreased. Moreover, ALP activity was enhanced in all tested cells except in U2OS cells upon 45S5 co-cultivation [137]. Despite the difference between the textural properties of conventional BG and MBG (high porosity and high surface area of MBG) that could enhance cytocompatibility, the cell type used could affect the biological behavior of tested glasses. In general, and according to the clinical application, the use of primary human cells represents the most suitable standard but has some limitations due to patient variability (sex, age, physiological conditions, etc.) which must be taken into consideration. However, the use of cell lines could be more advantageous regarding the reproducibility and the standardization of the experimental conditions resulting in comparable results obtained from different studies [138,139].

Only 41 articles (about 65% of the included studies) investigated cell morphology and/or the cell adhesion even though they are important parameters to evaluate biocompatibility. An appropriate choice of microscopy is relevant with SEM, CLSM, and fluorescence microscopy being well established in the field. However, optical techniques like optical imaging are often less precise and should be substituted or combined with one of the three techniques previously noted above. SEM allowed characterization of cell morphology and spreading, while CLSM allowed monitoring of cell colonization within scaffolds containing bioactive glasses. Some authors used more than one technique in order to provide different and complementary data.

Regarding cell viability or cell proliferation, 59 articles were found to have investigated them. Another relevant point is that, among these 59 articles, 36 confused the terms “cell viability” and “cell proliferation” and so that is why we had to treat both terms simultaneously in this review. Indeed, a cell viability test measures the ratio of living and dead cells in a population whereas a cell proliferation test assesses dividing cells [121]. Typically, using a LIVE/DEAD assay for cell proliferation is false, because it is only a cell viability test.

The different techniques used have all shown their efficiency for evaluating cell viability and proliferation. It is important to carefully choose the tests to be carried out, as there are many of them and each targets different cell processes. Trypan Blue, MTT, and LDH assays are the most common tests as they are inexpensive. When evaluating a new biomaterial, mostly reserved for bone regeneration, tests should be carried out over a comparatively longer timescale; a period of 28 days is usually required to cover the osteogenic differentiation needed to demonstrate bone remineralization, while shorter cultivation periods (1, 3, or 7 days) are long enough to reveal cytotoxicity when investigating both bone and soft tissue behavior. In this case, the Alamar Blue assay, which is non-toxic, highly sensitive, and stable, seems to be the best candidate test and could be more widely used in the future for biomaterials cytotoxicity assessment [140]. Moreover, some techniques such as the MTT and MTS assays can be qualitative and quantitative. That is why it is interesting to use them for both cell proliferation and viability. Both MTT and MTS are colorimetric tests and based on mitochondrial activity enzymes. However, the main difference between MTT, MTS, and WST-1 tests is that MTT assay has an additional step associated with the solubilization of formazan crystals whereas MTS and WST-1 assay are not associated with the solubilization of formazan crystals. The limitation of the MTT assay is that it requires the destruction of the cells for the analysis, thus making it impossible to use the cells for other investigations, following the same cell population within the same assay. It is also more time consuming [141].

Apoptosis quantification and cell cycle analysis are trials to study cell viability and death in more detail (three articles). Thanks to these tests, it is possible to have more precise information on the phases involved in cell death in contact with biomaterials, and therefore, on the cytotoxicity of the latter. Regarding the analysis of the cell cycle, this test is commonly performed using flow cytometry and consists of measuring the content of cellular DNA using a fluorescent dye that binds to DNA. Binding is quantified by measurement of the strength of the fluorescent signal. This DNA content finally makes it possible to differentiate between the different phases of the cell cycle [142]. Regarding the quantification of apoptosis, most of the time and from the included studies, this test is also carried out using flow cytometry through the analysis of DNA fragmentation [79,90,104].

**Figure 12:**
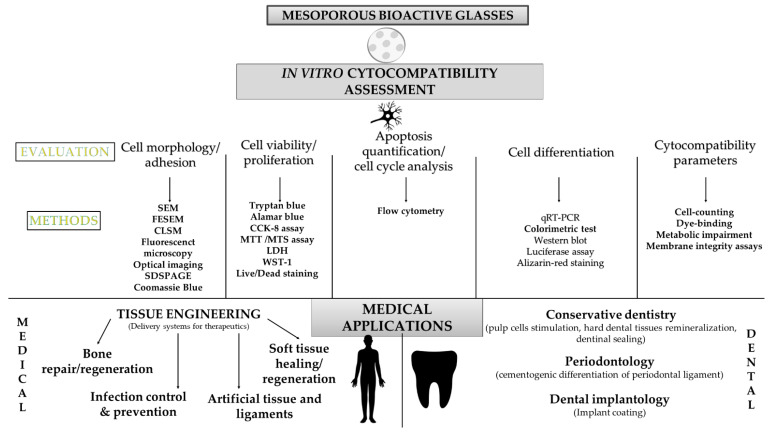
MBG cytocompatibility methodologies and their medical applications.

Forty-four articles (about 70%) investigated cell differentiation and 38 of them measured ALP activity. ALP is a marker found in all body tissues and the marker of early differentiation and extracellular matrix mineralization [143]. ALP activity revealed the differentiation potential of dental pulp cells after a short period (7 days) of contact with MBG [21]. In some articles, other markers were measured according to the target application as well as according to the cells studied (primary cells, established cell lines, cancer cells). The main markers used for bone regeneration were osteocalcin and osteopontin. Different angiogenesis factors such as VEGF were reported in these studies (Table 4). Figure 12 summarizes the methods used for the cytocompatibility assessment of mesoporous bioactive glasses and their medical applications.

A broad spectrum of techniques were found to used, that is to say colorimetric techniques, immunological techniques (ELISA, western blot), and qRT-PCR (Figure 12). However, western blot allowed a semi-quantification, unlike ELISA, that enabled a real quantification and should be privileged when possible. Combining qRT-PCR (gene expression) and ELISA (proteins level) could be relevant to provide a whole transcriptomic and proteomic data.

Four articles were excluded because they only explored biocompatibility in vivo. However, some studies show that in vitro tests are more effective in discriminating the cytotoxic nature of the material than in vivo tests [144]. The review of Keong et al. showed that testing in vitro was useful to characterize cytotoxic effects of molecules released from a biomaterial, such as residual monomers, catalysts, etc. [145]. This explains why they concluded that, when evaluating any newly developed biomaterial, in vitro tests should be conducted prior to in vivo tests in order to minimize the risk to humans and animals. Furthermore, we strongly believe that the use of predictive and standard in vitro tests could reduce the use of animal experiments, the scientific limits and societal impacts of which are highly recognized.

International organization for standardization (ISO) recommendations would help increase the repeatability and comparability of in vitro cytotoxicity and cytocompatibility studies. According to the ISO 10,993 recommendations regarding medical devices [123], one of the criteria for biocompatibility is the absence of material toxicity to cells. Cytotoxicity methods recommended by this norm and by the ISO norm 7405 [146] (which specifies test methods for medical devices used in dentistry biological effects evaluation) are cell-counting, dye-binding, metabolic impairment, or membrane integrity assays. The investigation of two parameters would be more reliable than the use of only one protocol to assess cytocompatibility. According to the critical review of Mocquot et al., bioactive glass bioactivity ranged from reactivity and apatite formation to pulp cell stimulation enhancement [9]. Even though “bioactivity assessment” is more demanding than cytocompatibility assessment when BG behavior is investigated in vitro, the link between the two assessments should be taken in consideration for comprehensive risk assessment of such materials.

## 5. Conclusions

Biocompatibility in vitro ranks as one of the most important properties to investigate with regards to the behavior of biomaterials, such as MBG. The articles investigated in this review clearly show the importance of validating the biocompatibility of these materials. However, there is a real lack of standardization regarding the methodologies used for MBG cytocompatibility assessment. For MBGs, primary cells seem to be the most appropriate cell model for studying biocompatibility. Microscopy is an essential technique for studying morphology and cell adhesion. Cell viability and proliferation, although often confused, can be investigated by a variety of relevant and predictive techniques. In some cases, it is interesting to go further by studying cytotoxicity in more depth by analyzing the cell cycle and quantifying apoptosis. Cell differentiation can be studied via the detection of certain precise markers by qRT-PCR which is the most frequently used and most precise technique. A multiparametric approach for all the tests (metabolic or not) could be interesting to improve the evaluation relevance and sensitivity. The standardization of all tests necessary to be performed to validate the biocompatibility of MBGs is a crucial point which will have to be further investigated in order to eliminate inconsistencies and, therefore, sources of error when comparing studies and unnecessary expenditure of time.

## Figures and Tables

**Table 1:** Research equations explored.

Keyword Selection	Number of Articles in PubMed
((mesoporous) AND (bioactive) AND (glass)) OR (MBG) AND (biocompatibility)	91
((mesoporous) AND (bioactive) AND (glass)) OR (MBG) AND (biocompatibility) AND ((dental) OR (tooth) OR (teeth))	8

**Table 2:** Articles excluded and their grounds for exclusion.

Articles	Grounds for Exclusion
Mao—2016 [22]	Only in vivo biocompatibility tests
Anand—2019 [23]	
Ghamor-Amegavi—2020 [24]	
Lalzawmliana—2019 [25]	
Li—2015 [26]	No biocompatibility data
Garg—2017 [27]	
Jiang—2017 [28]	
Shoaib—2017 [29]	
Baino—2018 [30]	
Baino—2018 [31]	
Fernando—2018 [32]	
Kargozar—2018 [33]	
Nawaz—2018 [34]	
Liu—2018 [35]	
Pourshahrestani—2019 [7]	
Mubina—2019 [36]	
Shadjou—2015 [37]	Review Articles
Baino—2016 [38]	
Galarraga-Vinueza—2017 [39]	
Vichery—2016 [40]	
Fiume—2018 [41]	
Kargozar—2018 [42]	
Kaya—2018 [43]	
Wu—2018 [44]	
Lalzawmliana—2020 [45]	
Gisbert-Garzarán—2020 [46]	
Sistanipour—2018 [47]	Articles not using mesoporous bioactive glasses [47,48]
Wu—2019 [48]	

**Table 3:** Articles included and details of each investigation.

References	Aims	MBG Composition	MBG Compared with Conventional BG?	MBG Elabos Method	Type of Cells Used	Culture Setting (Direct or Indirect Contact)	Techniques Used to Assess Biocompatibility	Main Results
Lin—2015 [49]	Bone regeneration	MBG (no information on the composition of the MBG) + polyglycerol sebacate (adjuvant)	No Blank control	Sol-gel	rBMSCs: rat bone marrow mesenchymal stem cells	Direct	Cell adhesion: Optical density Cell morphology: CLSM Cell viability: MTT assay Cell differentiation: ALP activity assay and qRT-PCR analysis (*RUNX-2, OCN, OPN*)	Same viability and morphology. Adhesion and proliferation increased with PGS concentration. At high concentrations, biocompatibility decreased due to acidification.
Min—2015 [50]	Bone regeneration	MPHS: MBG 80S15C (80SiO_2_: 15CaO: 5P_2_O_5_) + PHBHHx (adjuvant)	No Negative control (without treatment)	3D printing	hBMSCs: human bone marrow mesenchymal stem cells	Direct	Cell adhesion: SEM Cell viability: CCK-8 assay Cell differentiation: ALP activity assay and qRT-PCR analysis (*OCN, OPN*, *bFGF, SDF-1*)	Cell morphology: well-extended MPHS-1.0 and MPHS-1.5 with a higher DMOG enhanced cell % rate Higher ALP activity, expression levels of OCN and OPN than MPHS scaffolds.
Gómez-Cerezo—2016 [51]	Bone regeneration	MBG 58S (58 SiO_2_: 37 CaO: 5 P_2_O_5_) + polycapro-lactone (adjuvant)	No Polycaprolactone alone	Sol-gel	MC3T3-E1: murine osteoblastic cells	Direct	Cell morphology: SEM & CLSM Cell viability: Alamar Blue assay and membrane integrity: LDH: CytoTox-ONE ™ (Promega, G7890) Cell differentiation: ALP activity assay	MBG stimulated cell proliferation, colonization, and differentiation. Cell migration affected by architectural features and enhanced by the chemical release produced during the MBG dissolution.
Han—2016 [52]	Bone regeneration	MBG (no information on the composition of the MBG) + PMMA (adjuvant), titanium-doped	No Negative control: no titanium	Sol-gel	U2OS: human osteosarcoma cell line	Direct	Cell viability: MTT assay	Slight cytotoxicity for all titanium concentrations. No effect on the cell proliferation.
Hesaraki—2016 [53]	Bone regeneration	MBG 64S (64SiO_2_: 31CaO: 5P_2_O_5_) + resin poly-methacrylate (adjuvant)	No Resin alone	Sol-gel	Calvarium-derived newborn rat osteoblasts	Direct	Cell morphology: SEM Cell viability: MTT assay	Bioactive glass/resin composite biocompatible and osteoconductive.
Kim—2016 [54]	Bone regeneration	MBG 85S (85SiO_2_: 15CaO) functionalized with amino groups	No Blank control	Sol-gel	RAW264.7: murine macrophages	Direct	Cell morphology: CLSM Cell viability: CCK-8 assay Cell differentiation: qRT-PCR analysis (*c-fos, cathepsin-K, TRAP, NFATc1*)	Cell viability dose and time dependent. Morphological characteristics reflected results of cell viability.
Pourshahrestani—2016 [55]	Hemostasis	Ga-MBGs: MBG 80S15C (80SiO_2_: 15CaO: 5P_2_O_5_) doped with gallium Si/Ca/P/Ga: 80/15/5/0; 79/15/5/1; 78/15/5/2; 77/15/5/3	No Control: MBG without gallium	Sol-gel	HDFs: human dermal fibroblast cells	Indirect	Cell viability: MTT assay	All glasses were non-cytotoxic. Cell viability enhanced in the presence of 1% Ga-MBGs.
Singh—2016 [56]	Bone regeneration and drug delivery	MBG (no information on the composition of the MBG) doped C-dot	No Blank control	Sol-gel	HeLa cell line, MC3T3-E1 murine osteoblastic cells, rBMSCs: rat bone marrow mesenchymal stem cells	Direct	Cell viability: MTT assay and CCK-8 assay	High bioactivity in vitro and cell viability of the developed nanospheres, equivalent to the bioactive glass nanoparticles.
Tang—2016 [57]	Bone regeneration	TMS/rhBMP-2, TMS (trimodal MBG scaffold), MBG with different pore sizes (macro/micro/nano-porous) (no information on the composition of the MBG)	No Blank control	Sol-gel	rBMSCs: rat bone marrow mesenchymal stem cells and HUVECs: human umbilical vein endothelial cells	Direct	Cell morphology: CLSM & SEM Cell adhesion: CLSM Cell viability: LIVE/DEAD assay Cell differentiation: ALP activity assay and qRT-PCR analysis (*RUNX-2, OCN, OPN, BSP, GAPDH*)	Excellent cytocompatibility with all trimodal and bimodal scaffolds and desirable environment for cells attachment and colonization.
Vishnu Priya—2016 [58]	Bone regeneration	Hydrogel containing magnesium-doped bioglass (60SiO_2_: 30CaO:10MgO)	No Control: hydrogel without MBG	Sol-gel	HUVECs: human umbilical vein endothelial cells and ADSCs: rabbit adipose-derived stem cells	Direct	Cell proliferation: Alamar Blue Cell adhesion: Fluorescence microscopy Cell differentiation: ALP activity, immunofluorescence (ALP, OCN)	Hydrogels containing MBG showed early initiation of differentiation and higher expression of ALP and osteocalcin confirming the osteoinductive property of MBG.
Wang—2016 [20]	Bone regeneration	MBG 80S15C (80SiO_2_: 15CaO: 5P_2_O_5_) doped with copper Si/Ca/P/Cu: 78/15/5/2; 75/15/5/5	No Control: TCPS	Sol-gel	MC3T3: mouse fibroblast cells	Direct and indirect	Cell adhesion: SEM Cell viability: MTT assay and CLSM Cell differentiation: qRT-PCR analysis (*VEGF, bFGF* and *PDGF*)	Cytotoxicity of copper dose dependent. Copper: proangiogenic (promotes differentiation)
Wu—2016 [59]	Bone regeneration and osteoporosis	MBG 80S15C (80 SiO_2_:15CaO)	No Blank control	Sol-gel	rBMSCs: bone marrow mesenchymal stem cells derived from either sham control or ovariectomized (OVX) rats	Indirect	Cell proliferation: CCK-8 assay Cell morphology and cytoskeletal structure: fluorescence microscopy Cell differentiation: ALP staining, Alizarin Red S, Oil Red-O staining, qRT-PCR (*RUNX2, PPARγ, GAPDH*), western blot (WB) (Runx2, PPARγ, β-actin)	Lower concentration of MBG dissolution can promote osteogenesis but inhibit adipogenesis of the sham and OVX BMSCs.
Zhang—2016 [60]	Bone regeneration	Large-pore MBG (no information on the composition of the MBG)	No Blank control	Not indicated	ADSCs: rat adipose-derived stem cells	Direct	Cell morphology: SEM Cell viability: MTS assay Cell differentiation: qRT-PCR analysis (*ALP, OCN, OPG, PPAR gamma*)	Proliferation related to ions released. Large pore mesoporous glass promotes the expression of osteogenic-related genes but also inhibit the expression of adipogenic genes.
Zhang—2016 [61]	Bone regeneration	MBG 80S15C (80SiO_2_: 15CaO: 5P_2_O_5_) functionalized with amino groups	No Blank control	Sol-gel	Rabbit BMSCs: bone marrow mesenchymal stem cells	Direct	Cell adhesion: SEM Cell viability: MTT assay Cell differentiation: ALP activity assay & qRT-PCR analysis (*ALP, BSP, OCN, RUNX-2*)	Amino-MBGS: the most potent proliferative effect and the most effective osteoblastic differentiation potential.
Ge—2017 [62]	Bone regeneration	MBG on nanoTitanium film, doped with growth factor. Special composition related to doping: SiO_2_/CaO/P_2_O_5_/TiO_2_: 80/5/5/10	No Control without the drug	Sol-gel	rBMSCs: rat bone marrow mesenchymal stem cells	Direct	Cell differentiation: ALP activity assay and qRT-PCR analysis (*COL-1, OCN*)	Highest ALP activity and strong *Col-I* and *OCN* expressions on 200-MBG film cells: possibly, due to the surface of the glass that accelerates the signal transduction.
Kaur—2017 [63]	Bone regeneration	MBG 64S (64 SiO_2_: 31 CaO: 5 P_2_O_5_) doped with copper (2.5 to 10%)	No Blank control	Sol-gel	J774A.1: murine macrophage cell line	Direct	Cell viability: MTT assay and Membrane integrity: Trypan Blue assay	High concentrations of copper (from 1,95 µg/mL): toxic. With the decrease in concentration, all the MBGs increased live cell and decreased dead cell rates.
Li—2017 [64]	Gene delivery	MBG (no information on the composition of the MBG) + polyglycerol + Arg_8_ (to functionalize polymer), loaded with DNA	No Blank control	Sol-gel	Human HeLa cervical cancer cell line	Direct	Cell viability: LIVE/DEAD assay and CCK-8 assay	Good cell biocompatibility. Most cells in the complex-treated groups grew well in contact with the MBG/DOX-treated group.
Luo—2017 [65]	Bone regeneration	Nanofibrous MBG (no information on the composition of the MBG)	No Blank control	Sol-gel	Mouse osteoblasts	Direct	Cell morphology: SEM & Fluorescence microscopy Cell viability: LIVE/DEAD assay and CCK-8 assay Cell differentiation: ALP activity assay	60S40C scaffolds: favorable support for cell growth, proliferation, and differentiation.
Luo—2017 [66]	Bone regeneration	NanoMBG 58S (58 SiO_2_: 37 CaO: 5 P_2_O_5_)	No Blank control	Aerogel-based method	Primary mouse osteoblast cells	Direct	Cell morphology: Fluorescence microscopy Cell adhesion: Fluorescence microscopy Cell viability: CCK-8 assay Cell differentiation: ALP activity assay	58S scaffold: more cells growth and cell differentiation than scaffold without 58S.
Pourshahrestani—2017 [67]	Hemostasis	MBG 80S15C (80SiO_2_: 15CaO: 5P_2_O_5_) doped with gallium (Ga) + chitosan (CHT = adjuvant) Si/Ca/P/Ga: 79/15/5/1 Ga-MBG/CHT (wt.%): 10/90; 30/70; 50/50	No Negative control: chitosan alone	Sol-gel	HDFs: human dermal fibroblast cells	Direct	Cell viability: Alamar Blue assay and LIVE/DEAD assay Cell morphology: CLSM	Good biocompatibility. No cytotoxicity.
Qi—2017 [68]	Bone regeneration	MBG 80S (80SiO_2_: 16CaO: 4P_2_O_5_) + calcium sulfate hydrate (adjuvant)	No Control: calcium sulfate hydrate alone	3D printing	hBMSCs: human bone marrow mesenchymal stem cells	Direct	Cell adhesion: SEM Cell viability: CCK-8 assay Cell differentiation: ALP activity assay and qRT-PCR analysis (*ALP, OCN, OPN, RUNX-2*)	Cell viability, proliferation, and differentiation increased with increase MBG concentrations.
Sánchez-Salcedo—2017 [69]	Bone regeneration	MBG 75S (75SiO_2_: 20CaO: 5P_2_O_5_) & MBG 85S (85SiO_2_: 15CaO) both functionalized with amino groups or lysine	No Blank control	Sol-gel	MC3T3-E1: mouse osteoblastic cells	Direct	Cell morphology: Inverted optical microscopy Cell adhesion: Fluorescence microscopy Cell viability: MTS assay and membrane integrity: LDH: CytoTox-ONE ™ (Promega, G7890) Cell differentiation: ALP activity assay	In vitro cytocompatibility of MBGs was preserved functionalization.
Schumacher—2017 [70]	Bone regeneration and drug delivery	MBG 80S (80SiO_2_: 16CaO: 4P_2_O_5_) + CaP bone cement (calcium phosphate cement) (adjuvant)	No Control: calcium phosphate cement alone	Sol-gel	Saos2: human osteosarcoma cell line	Direct	Cell viability: WST-1	Higher cell adhesion and metabolic activity for composites compared to pure calcium cement.
Shoaib—2017 [71]	Bone regeneration	MBG (49SiO_2_: 20CaO: 20Na_2_O: 7K_2_O: 4P_2_O_5_ mol %) doped with potassium	Yes Control: Bioglass 45S5	Sol-gel	NHFB: normal human fibroblast cell line	Direct	Cell viability: CCK-8 assay Cell-cycle analysis: Flow cytometer Cell differentiation: ELISA (anti-OCN) and ALP activity assay	Cell viability: no differences. Cell-cycle analysis: MBG did not have any role in cell cycle dose-dependency.
Wang—2017 [72]	Bone regeneration	MBG (no information on the composition of the MBG) functionalized with amino groups	No Control: conventional MBG	Sol-gel	MC3T3-E1: mouse osteoblast cell line	Direct	Cell adhesion: CLSM Cell viability: CCK-8 assay Cell differentiation: ALP activity assay, Luciferase assay (*RUNX-2*) and qRT-PCR analysis (*GAPDH, OCN, OPN*)	Better cell adhesion with amino-MBGs than with conventional MBGs. Both MBGs with or without adjuvant promote osteoblastic differentiation.
Xin—2017 [73]	Bone regeneration	Nano MBG80S (80SiO_2_: 16CaO: 4P_2_O_5_) (MBGN) + methacrylicanhydride and gelatin (GelMA-G) (adjuvants)	No Blank control	Not indicated	MC3T3-E1: mouse osteoblast cell line	Direct and indirect	Cell adhesion: SEM Cell viability: CCK-8 assay Cell differentiation: ALP activity assay	GelMA-G-MBGN membrane enhanced osteogenesis differentiation.
Xue—2017 [74]	Bone regeneration	NanoMBG (NanoBGs) (No information on the composition of the MBG) loaded with miRNA (adjuvant)	No Blank control	Sol-gel	hBMSCs: human bone marrow mesenchymal stem cells	Direct	Cell morphology: Inverted fluorescent microscopy Cell adhesion: Inverted fluorescent microscopy Cell viability: Alamar Blue assay and LIVE/DEAD assay Cell differentiation: qRT-PCR analysis (*ALP, OPN, RUNX2*)	Good cell biocompatibility. NanoBGs could efficiently deliver miRNA to enhance osteogenic differentiation.
Yu—2017 [75]	Gene delivery	MBG 80S (80SiO_2_: 16CaO: 4P_2_O_5_) and silica nanoparticles	No Control: commercial transfection reagent PEI 25K and LIPO300	Sol-gel	hBMSCs: human bone marrow mesenchymal stem cells	Direct	Cell viability: LIVE/DEAD assay Cell differentiation: ALP activity assay	NanoBGs revealed significantly lower cytotoxicity than the commercial transfection reagents.
Cai—2018 [76]	Bone regeneration	MBG (no information on the composition of the MBG) + trapped BMP2 (adjuvant) + microspheres of chitosan (adjuvant) (containing IL8)	No Blank control	Sol-gel	rBMSCs: rat bone marrow mesenchymal stem cells	Direct	Cell viability: CCK-8 assay Cell differentiation: ALP activity assay and qRT-PCR analysis (*RUNX-2, COL-1, OPN, OCN, β-actin*)	Below 1 mg/mL, microspheres exhibited little cytotoxicity to rBMSCs.
Covarrubias—2018 [77]	Bone regeneration	NanoMBG (nBGs) (No information on the composition of the MBG) + chitosan/gelatin (CHT/Gel 1:1) (adjuvant)	Yes nMBG/CHT/Gel, and nBG/CHT/Gel bionano-composite scaffolds	Sol-gel	DPSCs: dental pulp stem cells	Direct	Cell viability: MTS assay Cell differentiation: ALP activity assay	Cell viability and proliferation decreased with the concentration of nBGs (due to high calcium release); 5% nBGs allowed the highest cell proliferation rate.
Fiorilli—2018 [78]	Bone regeneration	MBG 85S (85SiO_2_: 15CaO) doped with strontium	No Control: polystyrene	Sol-gel and aerosol-spray drying method	J774a.1, fibroblast cell line L929, Saos-2	Direct and indirect	Cell viability: MTT assay Cell differentiation: qRT PCR analysis (*ALP, COL-1, GAPDH, OPG, RANKL, SPARC*)	Good biocompatibility: reduction of the inflammatory response and stimulation of the pro-osteogenic genes’ expression.
Gómez-Cerezo—2018 [79]	Bone regeneration	MBG 75S (75SiO_2_: 20CaO: 5P_2_O_5_)	No Control: without material Then, different doses of MBG-75S (0.5 mg/mL, 1 mg/mL, 2 mg/mL)	Sol-gel	Human Saos-2, osteoclast-like cells, murine RAW 264.7 murine macrophages	Direct	Cell morphology: CLSM Cell viability: Membrane integrity: LDH: CytoTox-ONE ™ (Promega, G7890) Apoptosis quantification and cell-cycle analysis: Flow cytometry	Cytotoxicity is dose dependent. No inhibition of osteoclastogenesis. Decrease of resorption activity.
Hsu—2018 [80]	Bone regeneration	Apatite-modified MBG (no information on the composition of the MBG): MBGNFs (MBG nanofibers) with PMMA and sodium alginate (adjuvants)	No	Sol-gel	MG-63: human osteoblast-like	Direct	Cell morphology: Fluorescence microscopy Cell differentiation: Immunofluorescence (BSP and OCN)	Better cell adhesion but lower viability with macroporous microbeads containing MBG nanofibers than with glass beads.
Jia—2019 [81]	Osteoporosis	Sr-MBG (Porous strontium- incorporated mesopore- bioglass) MBG doped with 5% Sr	No Control: MBG 80S 80Si0_2_:15CaO:5 P_2_O_5_	Sol-gel	hPDLc: human periodontal ligament stem cells	Indirect	Cell differentiation: Remineralization (Alizarin Red staining and quantification), WB (hnRNPL, Setd2, H3K36me3, P-AKT, AKT, P-CREB) and RT qPCR analysis (*hnRNPL, Setd2, ALPL, RUNX-2, GAPDH*)	Sr-MBG scaffolds had visibly more new bone formation and vascular distribution in the healing area than MBG scaffolds. More frequent RUNX-2-positive cells were detected in the presence of Sr while the percentage of hnRNPL-positive cells was less in Sr-MBG group.
Kumar—2019 [82]	Bone regeneration	MBG 64S (64SiO_2_: 31CaO: 5P_2_O_5_) + surfactant	No No control at all	Sol-gel	Human Saos-2: Sarcoma osteogenic cells	Direct	Cell viability: Membrane integrity: LDH: CytoTox-ONE ™ (Promega, G7890)	Cell proliferation was significantly affected by the textural characteristics, which is related to dissolution rate of Ca, P, and Si.
Mandakhbayar–2019 [83]	Bone regeneration	Sr-doped (85SiO_2_:10CaO:5SrO) and Sr-free (85SiO_2_:15CaO) nanobioactive glasses	No Blank control	Sol-gel	DPSCs: dental pulp stem cells	Indirect	Cell cytotoxicity: WST-1, CCK-8 assay and CLSM Cell differentiation: ALP activity and Alizarin Red staining and quantification”	Sr-NBC: high bioactivity, excellent biodegradability, fast therapeutic ion release, and high drug loading capability, which potentiates its application in dentin−pulp complex regeneration therapy.
Pourshahrestani—2018 [84]	Hemostasis	Gallium-doped MBG (no information on the composition of the MBG)	No Control: other hemostatic reagents	Sol-gel	HDFs: human dermal fibroblast cells	Direct	Cell viability: MTT assay and LIVE/DEAD assay	No toxic effect. Significant increase of viability.
Qi—2018 [85]	Bone regeneration	3D printed borosilicate BG (6Na_2_O, 8K_2_O, 2MgO, 6SrO, 22CaO, 36B_2_O_3_, 18SiO_2_, 2P_2_O_5_; mol.%) coated with MBG (no information on the composition of the MBG)	Yes BG coated with MBG compared with BG without coating	3D printing	hBMSCs: human bone marrow mesenchymal stem cells	Direct	Cell adhesion: SEM Cell viability: CCK-8 assay Cell differentiation: ALP activity assay and qRT-PCR analysis (*RUNX-2, OCN, COL1*)	Well-spread cell morphology. Proliferation rates of BG-MBG > BG scaffolds (*P* < 0.05). ALP activity: BG-MBG > BG (*P* < 0.05). Upregulation of osteogenic-related genes in cells grown on BG-MBG (*P* < 0.05).
Shoaib—2018 [86]	Drug delivery	MBG doped with potassium (special composition related to doping: 49SiO_2_: 20CaO: 20Na_2_O: 7K_2_O: 4P_2_O_5_ mol %) with variable percentages was used as filler in arginine and starch-based PU matrices (adjuvant)	No Different percentages of MBG in the nanocomposite	Sol-gel	NHFB: normal human fibroblast cell line	Direct	Cell viability: MTT assay	Enhancement of cell adhesion. No significant difference on cell viability.
Zeng—2018 [87]	Bone regeneration	MBG 80S (80SiO_2_: 16CaO: 4P_2_O_5_) functionalized with amino groups (N-MBG)	No Blank control	Not indicated	Rabbit BMSCs: bone marrow mesenchymal stem cells	Direct and indirect	Cell viability: LIVE/DEAD assay and MTT assay Cell differentiation: ALP activity assay and qRT-PCR analysis (*CaSR, RUNX-2, GAPDH, OPG, RANKL, IL-10, Arg-1*)	Better cell viability and differentiation with N-MBG. Decreased gene expression after NPS2143 (CaSR signaling pathway inhibitor) treatment.
Du—2019 [88]	Bone regeneration	MBG (no information on the composition of the MBG) + silk fibroin (adjuvant) (MBG/SF)	No Control: MBG/PCL scaffold	Sol-gel	hBMSCs: human bone marrow mesenchymal stem cells	Direct	Cell adhesion: SEM and CLSM Cell viability: CCK-8 assay Cell differentiation: ALP activity assay and qRT-PCR analysis (*BMP-2, OCN, OPN, BSP, COL-1*)	Better cell adhesion and proliferation on MBG/SF composite scaffold.
Fu—2019 [89]	Drug delivery	MBG (no information on the composition of the MBG) + sodium alginate (SA) (adjuvant)	No SA alone	3D printing	hBMSCs: human bone marrow mesenchymal stem cells	Direct	Cell adhesion: CLSM Cell viability: CCK-8 assay Cell differentiation: ALP activity assay and qRT-PCR analysis (*OCN, COL-1, BMP-2, BSP*)	More live cells, better cell proliferation and differentiation were observed on MBG/SA and MBG/SA–SA scaffolds compared to SA scaffolds.
Gómez-Cerezo—2019 [90]	Osteoporosis, bone regeneration and drug delivery	MBG58S (58 SiO_2_: 37 CaO: 5 P_2_O_5_) + e-polycapro-lactone (adjuvant)	No Blank control	Sol-gel	Human Saos-2 osteoblasts, RAW-264.7 murine macrophages	Direct and indirect	Cell morphology: CLSM Apoptosis quantification: Flow cytometer Cell-cycle analysis: Flow cytometer	Zoledronic acid released from MBG produced osteoblast apoptosis and a delay of osteoblast proliferation in a time-dependent way; caused an inflammation and fibrosis.
Li—2019 [91]	Bone regeneration and drug delivery	Scaffold PLGA- MBG (SiO_2_-CaO-P_2_O_5_, Si/Ca/P=80:15:5) and FTY/MBG-PLGA FTY (adjuvant)	No Control: PLGA without MBG	Sol-gel	rBMSCs: rat bone marrow mesenchymal stem cells and HUVECs: human umbilical vein endothelial cells	Indirect	Cell adhesion: Immunofluorescence Cell proliferation: CCK-8 assay Cell-cycle analysis: WB (Erk1/2, p-Erk1/2) Cell differentiation: ALP activity assay, Alizarin Red S, crystal violet, immunofluorescence (OCN), qRT-PCR analysis (*β-actin, ALP, OCN, BMP-2, Osterix, Hif-1α, VEGF-A, CXCR4*), WB (Hif-1α and β-actin)	The scaffolds exhibited sustained release of the bioactive lipids as well as the calcium and silicon ions, which were demonstrated to broadly enhance biological activities including the adhesion, proliferation, and osteogenic differentiation of rBMSCs as well as the proliferative and in vitro angiogenic ability of HUVECs.
Liu—2019 [92]	Bone regeneration	MBG (no information on the composition of the MBG)-hydroxyapatite + silk fibroin (adjuvant)	No Silk fibroin alone	Sol-gel	hMSCs: human mesenchymal stem cells	Direct	Cell morphology: FESEM (field emission scanning electron microscopy) Cell viability: MTT assay Cell differentiation: ALP activity assay and qRT-PCR analysis (*COL-1, OCN, RUNX-2, GAPDH*)	Positive osteogenic differentiation effect and upregulated osteoblastic gene expression of samples containing a high concentration of hydroxyapatite.
Montalbano—2019 [93]	Bone regeneration	NanoMBG_Sr4% (dopant) + Type I collagen (adjuvant), with 4-StarPEG crosslinked special composition of MBG related to doping (Sr/Ca/Si = 4/11/85 %mol)	No Control: TCPS and non-crosslinked biomaterial samples	Sol-gel	MG-63 human osteoblast-like	Direct and indirect	Cell adhesion: SEM Cell viability: Alamar Blue assay and indirect cytotoxicity assay using “conditioned medium”	Good cell adhesion, morphology, and viability.
Pourshahrestani—2019 [94]	Bone regeneration	AgMBG/POC: silver-doped MBG 80S15C (80SiO_2_: 15CaO: 5P_2_O_5_) + poly (1,8 octanediol citrate) (adjuvant) Si/Ca/P/Ag: 79/15/5/1: 1%AgMBG AgMBG/POC (wt.%): 5/95; 10/90; 20/80	No Control: adjuvant alone	Sol-gel	HDFs: human dermal fibroblast cells	Direct	Cell viability: Alamar Blue assay	Efficient antibacterial properties while preserving a favorable biocompatibility.
Terzopoulou—2019 [95]	Bone regeneration	MBG 80S (80SiO_2_: 16CaO: 4P_2_O_5_) doped with calcium or strontium + polycapro-lactone (PCL) (adjuvant)	No Blank control	Sol-gel	WJ-MSCs: human Wharton’s jelly-derived mesenchymal stem cells	Indirect	Cell adhesion: Fluorescence microscopy Cell viability: MTT assay	No cytotoxicity observed after 24 h. Osteoinductive additives in PCL matrices facilitate the differentiation.
Varini—2019 [96]	Bone regeneration	Cerium-doped (0 to 5%) MBG 80S (80SiO_2_: 16CaO: 4P_2_O_5_) + sodium alginate (adjuvant)	No Blank control	Sol-gel	MC3T3-E1: mouse osteoblastic cells	Direct and indirect	Cell viability: Alamar Blue assay and membrane integrity: LDH: CytoTox-ONE ™ (Promega, G7890) Cell differentiation: ALP activity assay	Better proliferation. No release of cytotoxic agent. Differentiation decreased with the amount of cerium.
Wang—2019 [17]	Bone regeneration	MBG and MBG-L (larger pores) (no information on the composition of the MBG), FGF (fibroblast growth factor) adsorbed (adjuvant)	No Control: simple MBG	Sol-gel	MC3T3-E1: mouse osteoblast cell line	Direct	Cell adhesion: laser microscope Cell viability: CCK-8 assay Cell differentiation: ALP activity assay, Luciferase assay (*RUNX-2*) and qRT-PCR analysis (*RUNX-2, OCN, OPN, GAPDH*)	Better cell adhesion, proliferation, differentiation with large-pore MBG. Adding FGF enhanced the cell adhesion and differentiation even more.
Wang—2019 [97]	Bone regeneration	MBG80S15C (80SiO_2_: 15CaO: 5P_2_O_5_) + GO (graphene oxide) (adjuvant)	No Control: MBG alone	Sol-gel	rBMSCs: rat bone marrow mesenchymal stem cells	Direct and indirect	Cell morphology: SEM Cell adhesion: SEM Cell viability: CCK-8 assay Cell differentiation: ALP activity assay, RT-qPCR analysis (*RUNX-2, ALP, OCN, COL-1, VEGF, HIF-1α*) and immunofluorescence (OCN)	Better cell proliferation and differentiation with the MBG containing an adjuvant.
Wu—2019 [98]	Bone regeneration in osteoporosis	MBG 80S (80SiO_2_: 16CaO: 4P_2_O_5_) + sodium alginate (SA) + gelatin (G); soaked with calcitonin gene-related peptide (CGRP) or Naringin (NG) (adjuvants)	No Control: MBG and MBG/SA/G	Sol-gel	MG-63: human osteoblast-like	Direct and indirect	Cell adhesion: Inverted fluorescent microscopy and SEM Cell viability: CCK-8 assay Cell differentiation: qRT-PCR analysis (*RUNX-2, ALP, OPN, OCN*)	No difference in adhesion. Drug adjuvants enhanced proliferation and differentiation.
Zhang—2019 [99]	Bone regeneration	MBG (no information on the composition of the MBG)	No Blank control	Not indicated	rBMSCs: rat bone marrow mesenchymal stem cells	Direct	Cell morphology: SEM and TEM Cell proliferation: Immunofluorescence (Ki67) Cell differentiation: WB (ALP, RUNX-2, OCN, BMP-2, β-actin, Gli1, Smo), qRT-PCR analysis (*ALP, RUNX-2, OCN, BMP-2, Gli1, Smo*) and RNA	Bioactive glass–ceramic coating promoted proliferation and differentiation, and up regulated the expression of osteogenesis-related genes.
Zheng—2019 [100]	Bone regeneration, drug delivery	Cu-MBGNs: MBG 85S (85SiO_2_: 15CaO) doped with copper (0 to 10%)	No Blank control	Sol-gel	Mesenchymal stromal ST2 cells derived from mouse bone marrow of BC8 mice	Indirect	Cell viability: CCK-8 assay	Cytotoxicity of Cu-MBGNs was related to the concentration of Cu ions as well as the dosage of particles applied.
Berkmann—2020 [101]	Bone regeneration	MBG 85S(85SiO_2_: 15CaO)	No Blank control	Aerosol spray-drying method	hMSCs: human mesenchymal stem cells	Indirect	Cell viability: PrestoBlue, LDH Cell counting: DAPI test. Cell differentiation: Alizarin-Red staining	Ionic dissolution products amplify the osteogenic differentiation of hMSCs.
Chitra—2020 [102]	Bone regeneration	MBG 45S5 (45SiO_2_: 24.5CaO: 24.5Na_2_O: 6P_2_O_5_): crystalline phase: Na_2_Ca_2_Si_3_O_9_ and Na_2_Ca_3_Si_6_O_16_	Yes	Sol-gel	MG-63: human osteoblast-like and PBMC: human peripheral blood mononuclear cell	Direct	Cell viability: MTT assay	The probe sonication enriches the biocompatibility.
Mocquot—2020 [21]	Bone regeneration	MBG 75S(75SiO_2_: 15CaO: 10P_2_O_5_)	No Blank control	Sol-gel	hDPCs: human dental pulp cells	Indirect	Cell morphology: CLSM Cell viability: Alamar Blue assay Cytotoxicity: crystal violet test Cell differentiation: ALP activity assay and immunofluorescence (OCN and DMP-1)	The MBG showed no cytotoxicity, good cytoskeletal architecture, cell spreading and adhesion.
Montalbano—2020 [103]	Osteoporosis	Nano MBG (85% SiO_2_: 11% CaO + 4% Sr) + collagen (adjuvant)	No Blank control	Sol-gel	MG-63: human osteoblast-like + SaOS-2: human osteosarcoma cell line	Direct	Cell viability: Alamar Blue assay Cell adhesion and morphology: SEM	The developed hybrid system largely proved its biocompatibility in presence of MG-63 and Saos-2 cells.
Montes-Casado—2020 [104]	Immunity	NanoMBG (81.44% SiO2- 18.6% CaO)	No Control: cells without NanoMBGs	Sol-gel	Murine BMDCs: Bone marrow-derived dendritic cells + SR.D10 Th2 CD4+ lymphocytes + DC2.4 dendritic cells	Direct	Cell differentiation: Flow cytometry assays (FACS + antigenic markers) Cell inflammation: cytokine expression: immunofluorescence (FACS + cytokine markers) Cell proliferation: CellTraceTM Violet dye (FACS) Apoptosis: Annexin V (FACS) Cytotoxicity: CLSM	NanoMBGs were both non-toxic and non-inflammagenic for murine lymphoid cells and myeloid DCs despite their efficient intake by the cells.
Pontremoli—2020 [105]	Bon regeneration	Zwitterionic or no zwitterionic Nano (_SG) and micro (_SD) particles of MBG_Sr2% (85SiO_2_:13CaO:2Sr)	No Control: cells without MBGs	Sol-gel	MC3T3-E1: mouse osteoblast cell line	Direct	Cell proliferation: MMT-test Cytotoxicity: LDH Cell differentiation: Alizarin Red staining and quantification Cell adhesion: SPS-PAGE and Coomassie blue for visualization	After zwitterionization the in vitro bioactivity was maintained, no cytotoxicity about Sr-MBG particles. Zwitterionic Sr-MBGs showed a significant reduction of adhesion.
Wang—2020 [106]	Bone regeneration	Large-pore MBG (no information on the composition of the MBG) + genistein (adjuvant)	No Control: MBG with normal pore size	Sol-gel	MC3T3-E1: mouse osteoblast cell line	Direct	Cell adhesion: Fluorescence microscopy Cell viability: CCK-8 assay Cell differentiation: ALP activity assay and qRT-PCR analysis (*OPN, GAPDH*)	Genistein is a molecule good for cell attachment. MBG-L/G is the better substrate for osteoblast differentiation.
Zhou—2020 [107]	Bone regeneration	MBG/SIS-P28: MBG (no information on the composition of the MBG) doped with SIS (porcine small intestinal submucosa) + BMP2-related peptide P28 MBG/SIS-H-P28 (heparinized MBG/SIS)	No Control: MBG/SIS	Sol-gel	MC3T3-E1: mouse osteoblast cell line	Direct	Cell proliferation: MTT-test Cell viability: CLSM Cell differentiation: ALP activity, Alizarin Red staining) and qRT-PCR analysis (*GAPDH, RUNX-2, OCN, OPN, ALP*)	MBG/SIS-H-P28 scaffolds exhibit a much stronger ability to stimulate bone regeneration.
Zhou—2021 [108]	Bone regeneration	MBGNs (no information on the composition of the mesoporous bioactive glass nanoparticles)	No Control: gelatin (Gel)/oxidized chondroitin sulfate (OCS) hydrogel without MBGN + different MBGN concentrations (0%, 5%, 10% and 15%)	Sol-gel	rBMSCs: rat bone marrow mesenchymal stem cells	Direct	Cell adhesion and spreading: CLSM Cell proliferation: CCK-8 assay Cell viability: LIVE/DEAD assay Cell differentiation: Immunostaining (RUNX-2), ALP activity assay, qRT-PCR analysis (*OCN, RUNX-2, OPN, COL-1*), WB (OCN, OPN), Immunofluorescence (OPN) and Alizarin Red staining	Presence of MBGNs enhanced proliferation of BMSCs and osteogenic differentiation of BMSCs grown on Gel/OCS/MBGN hydrogel surfaces.

Abbreviations: PGS (poly(glycerol sebacate); PHBHHx (poly(3-hydroxybutyrate-co-3-hydroxyhexanoate)); DMOG (dimethyloxallyl glycine); LDH (lactate dehydrogenase); PMMA (poly(methyl methacrylate); TRAP (tartrate-resistant acid phosphatase), NFATc1 (nuclear factor of activated T-cells cytoplasmic 1); C-dot (carbon dot); rhBMP-2 (recombinant human bone morphogenetic protein-2); TCPS (tissue culture polystyrene); VEGF (vascular endothelial growth factor); PDGF (Platelet Derived Growth Factor); PPAR-gamma (peroxisome proliferator-activated receptor gamma); Arg_8_ (octoarginine); DOX (doxorubicin); RANKL (receptor activator of nuclear factor kappa-Β ligand); SPARC (secreted protein, acidic, cysteine-rich); hnRNPL (heterogeneous nuclear ribonucleoprotein L); Setd2 (SET domain containing 2); P-AKT (phosphorylated Akt); Akt (protein kinase B); P-CREB (phospho-cAMP response element-binding protein); PU (polyurethanes); CaSR (calcium-sensing receptor); BMP-2 (bone morphogenetic protein-2); PLGA (particle-poly (lactic-co-glycolic acid); FTY (Fingolimod); Erk_1/2_ (extracellular signal-regulated kinase _1/2_); p-Erk_1/2_ (phospho-Erk_1/2_); 4-StarPEG (4-star poly(ethylene glycol) ether tetrasuccinimidyl glutarate); HIF-1α (hypoxia-inducible factor-1α); Hh (hedgehog); Gli1 (glioma-associated oncogene); Smo (smoothened); DMP-1 (dentin matrix acidic phosphoprotein 1); FACS (fluorescence-activated cell sorting for flow cytometry).

**Table 4:** Main cell differentiation markers and their application.

Topic of the Article	Studied Markers
Bone regeneration	Osteocalcin (OCN), runt-related transcription factor 2 (RUNX-2), osteopontin (OPN), bone sialoprotein (BSP), osteoprotegerin (OPG)
Angiogenesis	Vascular endothelial growth factor (VEGF), hypoxia-inducible factor 1-alpha (HIF1A), basic fibroblast growth factor (bFGF), stromal-derived factor 1 (SDF-1)

## Data Availability

Not applicable.
